# Reimagining peer review as an expert elicitation process

**DOI:** 10.1186/s13104-022-06016-0

**Published:** 2022-04-05

**Authors:** Alexandru Marcoci, Ans Vercammen, Martin Bush, Daniel G. Hamilton, Anca Hanea, Victoria Hemming, Bonnie C. Wintle, Mark Burgman, Fiona Fidler

**Affiliations:** 1 Centre for Argument Technology, School of Science and Engineering (Computing), University of Dundee, Dundee, UK; 2 School of Communication and Arts, The University of Queensland, Brisbane, QLD Australia; 3 MetaMelb Lab, University of Melbourne, Melbourne, VIC Australia; 4 Centre of Excellence for Biosecurity Risk Analysis, University of Melbourne, Melbourne, VIC Australia; 5 Martin Conservation Decisions Lab, Department of Forest and Conservation Sciences, University of British Columbia, Vancouver, Canada; 6 Centre for Environmental Policy, Imperial College London, London, UK

**Keywords:** Peer review, Expert elicitation, Wisdom of the crowd, Anonymity, DELPHI

## Abstract

Journal peer review regulates the flow of ideas through an academic discipline and thus has the power to shape what a research community knows, actively investigates, and recommends to policymakers and the wider public. We might assume that editors can identify the ‘best’ experts and rely on them for peer review. But decades of research on both expert decision-making and peer review suggests they cannot. In the absence of a clear criterion for demarcating reliable, insightful, and accurate expert assessors of research quality, the best safeguard against unwanted biases and uneven power distributions is to introduce greater transparency and structure into the process. This paper argues that peer review would therefore benefit from applying a series of evidence-based recommendations from the empirical literature on structured expert elicitation. We highlight individual and group characteristics that contribute to higher quality judgements, and elements of elicitation protocols that reduce bias, promote constructive discussion, and enable opinions to be objectively and transparently aggregated.

## Introduction

Trust in the good judgement of reviewers with relevant qualifications, experience, and scientific skill is at the heart of peer review. Editors value reviewers’ expertise, and multiple studies have found a strong correlation between editorial final decisions and reviewers’ judgements [1–3]. However, human judgement (even experts’) is often flawed, susceptible to conscious and unconscious prejudices, misunderstandings, and gaps in knowledge. It should therefore be unsurprising that peer review can also be biased [4]. Peer reviewers have been shown to overlook methodological flaws and statistical errors [5–7], avoid reporting suspected instances of fraud [8] and commonly reach a level of agreement barely exceeding what would be expected by chance [9]. Recent studies have also exposed the extent of gender bias in peer review [10] and questionable editorial protocols that lack transparency [11]. Despite the wide range of issues, the debate over whether the system is irrevocably broken has not been settled. What is clear is that more work is needed to understand how journal peer review functions, to identify pressure points and improve its efficacy as a gatekeeping mechanism for high quality science [12].

Contemporary peer review is predominantly a journal-organised, pre-publication quality assurance activity wherein independent and (often) anonymous reviewers provide their opinion on the suitability of submissions for publication [11, 13, 14], with reviewers being prompted with open questions or with several criteria that they should consider when making judgements [14]. Over the last two decades, editorial procedures have begun gradually to diverge from these conventional features, although the advances are slow and subject to criticism about time delays and undue influence over the process [13, 15]. A small minority of journals are experimenting with innovative peer review models that encourage dialogue between invited reviewers (between 2 and 8% of journals) [11, 14, 16]. While these more collaborative approaches are a promising development, they have not solved critical issues around limited participation, reviewer coherence or accountability. We believe that additional structural changes are required. To provide actionable recommendations for a fairer, accountable and more transparent system, we start from the observation that peer review should be treated like a structured expert elicitation process, applying tested protocols that incorporate research from mathematics, psychology, and decision theory to mitigate biases, and enhance the transparency, accuracy, and defensibility of the resulting judgements. This can demonstrably improve the quality of expert judgements, especially in the context of critical decisions [17–21]. In what follows we outline the hypothetical benefits of applying structured expert elicitation principles in journal peer review. In the Outlook section, we reflect on the challenges ahead.

## Main text

### Peer review as a structured elicitation process

**Fig. 1:**
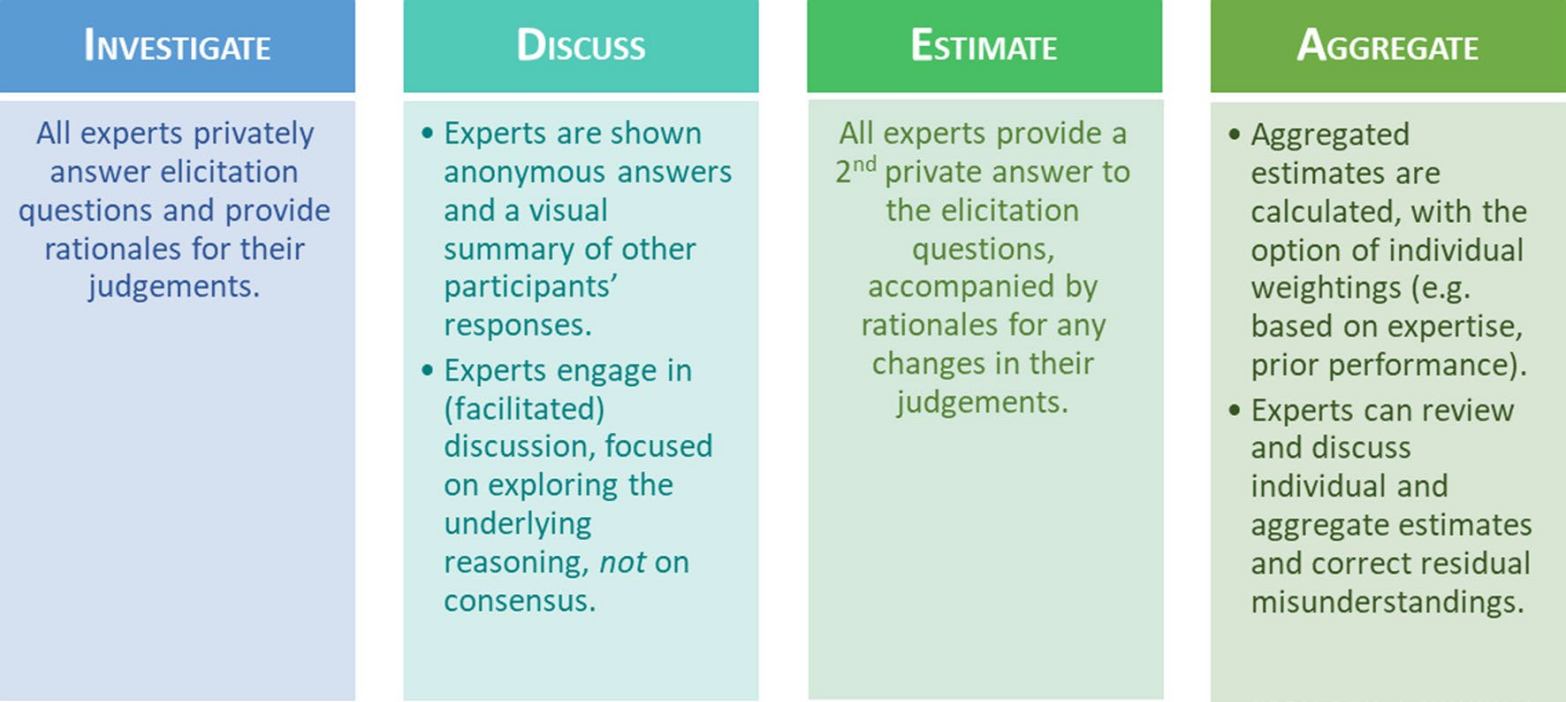
The IDEA protocol for structured expert judgement elicitation (adapted from [20])

Our recommendations are based on our collective experience developing and implementing the IDEA protocol (Investigate—Discuss—Estimate—Aggregate, Fig. 1) for structured expert elicitation in diverse settings including conservation, intelligence analysis, biosecurity, and, most recently, for the collaborative evaluation of research replicability and credibility [22–28]. This Delphi-style protocol has been shown to facilitate accurate predictions about which research findings will replicate [29] by prompting experts to investigate and discuss the transparency and robustness of the findings in a structured manner.

**Fig. 2:**
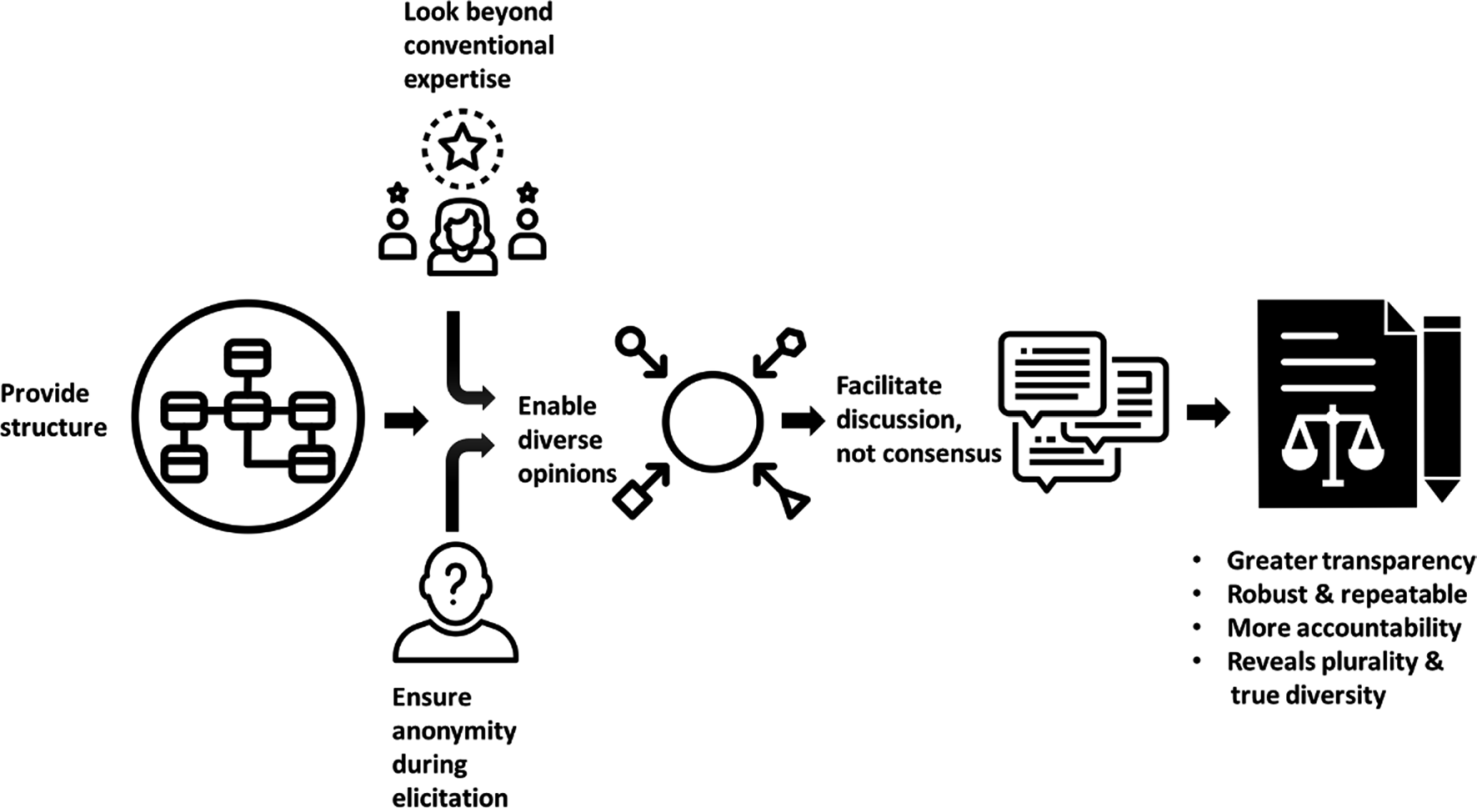
A reimagined peer review process

In the following sections, we outline five recommendations focusing on individual and group characteristics that contribute to higher quality judgements, and on ways of structuring elicitation protocols that promote constructive discussion to enable editorial decisions that represent a transparent aggregation of diverse opinions (Fig. 2).

#### Elicit diverse opinions

One of the crucial phenomena that underpins most expert elicitation protocols is the wisdom of the crowd effect [30], a statistical phenomenon where random errors associated with independent judgements cancel each other out, driving collective judgement closer to the truth [31–34]. In quantitative and probabilistic judgements, groups of diverse individuals often perform as well as or better than even the best-credentialed single expert [35, 36].

In the context of peer review, diverse experiences and worldviews may provide different perspectives and judgements on a manuscript [1, 37, 38]. This is particularly important because perceptions about the quality of new research are inherently subjective and socially constructed [39–41]. Perhaps for these reasons, reviewers have been shown to favour manuscripts on topics familiar to them [42, 43], and to undervalue manuscripts reporting results that contradict their prior beliefs [44] or written by members of traditionally underrepresented groups in academia [45, 46].

Editors will typically attempt to recruit reviewers who collectively cover the knowledge space corresponding to the topic of a paper, as would be advised in expert elicitations [47, 48]. This process, however, is often subject to bias. Recent research shows, for instance, that female reviewers have fewer opportunities to participate in peer review [49]. There is already ample discussion of the need to increase diversity and inclusion in peer review, but we argue that adopting more inclusive practices in peer review has benefits beyond achieving better representation. Demographic diversity (i.e., variation in age, gender, cultural background, life experience, education and specialization) can serve as a pragmatic proxy for the more crucial characteristic of cognitive diversity that underpins the wisdom of the crowd effect [50].

However, leveraging the collective insight of a diverse crowd of reviewers will not necessarily make the recruitment of peer reviewers more difficult. Most structured expert elicitation processes use between 3 and 7 experts. A recent survey of peer reviewing practices in 142 journals from 12 different disciplines and comprising 100,000 papers uncovered that on average each paper received 3.49 ± 1.45 (SD) reviews [51]. Accessing diverse “experts” may nevertheless be challenging, particularly in small, specialised fields, and require alternative approaches (e.g., recruiting graduate students, researchers in third sector organisations, government agencies, and industry), which leads us to our second recommendation.

#### Challenge conventional definitions of expertise

We tend to credit people with expertise based on their status, publications, affiliation with well-known institutions etc., yet such credentials have been shown to be unreliable criteria. Studies have shown mixed results for the association between traditional markers of expertise and indicators of judgement performance, such as overconfidence [52], reliability [53], calibration [54], and coherence [55]. Furthermore, selecting experts using conventional criteria can often bias the demographics of experts towards older individuals, and often males [28, 53]. To foster diversity, we must challenge our definition of expertise. Instead of setting our sights on a small population of narrowly defined “experts”, our focus should be on engaging the wider scientific community, aiming to build skillsets in judging the quality of academic outputs through ‘deliberate practice’ [56, 57], which is a more relevant definition of expertise in this context.

Several large-scale projects have shown peer-reviewed research in medicine, psychology and economics often fails to replicate [58–63], raising fundamental questions about the scientific process, including peer reviewers’ abilities to detect valid and reliable research. Yet recent studies have shown that, under the right conditions, groups of laypeople can accurately predict which research claims will replicate [64]. A computational analysis of the peer review process suggests “the accuracy of public reader-reviewers can surpass that of a small group of expert reviewers if the group of public reviewers is of sufficient size” [65]. Therefore, we argue that rather than relying on conventional expertise, the aggregate of judgements of groups of assessors with diverse knowledge, drawn from traditional and non-traditional reviewer pools, will result in more accurate judgements. Widening the potential reviewer pool may therefore convey benefits on review quality, in addition to addressing crucial ethical (i.e., increasing diversity and inclusion) and pragmatic concerns (i.e., reducing the burden on an already strained minority that generate the bulk of peer reviews [66]).

#### Provide structure

One aim of structuring elicitation processes is to quantify (but not eliminate) uncertainty. Structured expert elicitation protocols achieve this by standardising the response formats of quantitative elicitation questions, and IDEA, for instance, asks experts to provide bounds that give a measure of uncertainty. The procedure removes consensus pressure and associated biases by aggregating individual judgements mathematically rather than behaviourally. In peer review, this can be achieved by structuring judgements around a predefined set of questions about research quality, expressed in quantitative terms (some peer review rubrics do this already [67]).

Importantly, many structured expert elicitation protocols complement quantitative estimation in several ways, i.e. by collecting written feedback, facilitating discussion among experts, encouraging experts to correct misunderstandings, and by exploring diverse perspectives and counterfactual evidence without forcing consensus. A peer review process modelled after the IDEA protocol will generate both quantitative and qualitative data that will feed into editorial decision-making.

Nevertheless, the use of numerical scores may give an unwarranted impression of precision. Indeed, the extent to which different reviewers and/or the editors share conceptualisations or reference points for these scores is questionable and peer reviewers often are miscalibrated [68–70]. Notwithstanding these legitimate concerns, asking peer reviewers to provide numerical scores and interval judgements, in addition to narrative evaluations, may more readily highlight areas of disagreement and uncertainty. In discussion, expert groups may resolve some of their initial disagreements, while remaining disagreements may require the attention of the editor(s) and/or author(s).

#### Encourage and facilitate interaction

When faced with uncertainty, individuals – including experts – use heuristics, or cognitive shortcuts, that may lead to erroneous conclusions [76]. Group interaction increases the probability of identification and subsequent correction of errors [77] and can counter individual cognitive biases [78]. Interaction among experts also has a synergistic effect and may generate novel ideas and solutions that would not have been produced individually [71–75]. Nevertheless, the intellectual gain of group interaction is moderated by coordination costs [79], overconfidence [53, 80, 81] and deference to high-status or particularly vocal group members [82]. Expert elicitation protocols mitigate many of these risks by supporting experts to investigate and discuss the rationales that underpin individual judgements. The process aims to develop more comprehensive, unprejudiced understandings and create shared mental models [83]. By explicitly revealing private information, the process attempts to mitigate the pernicious effects of unshared information [84, 85] and prompts experts to imagine alternative realities, which reduces subjectivity and (over)confidence [86].

Applied to peer review, the interactive process we envision encourages reviewers to articulate their reasoning explicitly before sharing it with others, and subsequently to probe the rationales that underpin alternative judgments. It also promotes the resolution of conflicts due to misunderstandings, lack of relevant knowledge, or the application of idiosyncratic evaluation criteria [44, 87–90]. Importantly, it does not force consensus where true agreement does not exist. From the editor’s point of view, having access to both outcome (individual reviewer decisions) and process data (interaction among reviewers) generates valuable insights into how reviewers’ rationales withstand the scrutiny of their peers, distinguishes between trivial and fundamental differences of opinion, and ultimately enables a more informed and transparent editorial decision.

While interactions among reviewers are still relatively uncommon in journal peer review, interviews with editors and reviewers uncovered a practice of conferring with colleagues on how to review a manuscript and collective decision-making [91]. This suggests that both editors and peer reviewers may welcome opportunities to make the process more interactive.

#### Anonymise judgements

To further mitigate potential pernicious social influences that can undermine the wisdom of crowd [82], it is important to maintain some degree of independence, to enable individuals to express their true beliefs, without dominance or interpersonal conflict. Expert elicitation protocols like IDEA protect the anonymity of judgements [28] and encourage experts to evaluate each idea on its own merit, rather than on the status of its proponent, thus minimising some (although arguably not all) social biases.

Anonymity is a contested feature of traditional peer review protocols. Critics claim that it encourages laziness, idiosyncratic (or self-interested) reviews, responsibility and even abuses of power [91, 92]. Opening reviewers’ judgments to the scrutiny of their peers and/or editors would mitigate some of these dangers or at least expose them before they are sent to authors and become enshrined in editorial decisions. What is more, reviewers’ identities could also be revealed at the end of the elicitation process. So, by combining anonymous judgements with group interaction, the re-imagined peer review process outlined in this paper preserves the advantages of the traditional peer review system and mitigates the danger of unaccountable reviewers.

### Outlook

Efforts are underway to support the implementation of more transparent peer-reviewing practices [93–95]. This commentary contributes to a wider conversation about establishing peer review processes grounded in evidence-based practice. Our suggestions are based on observations about how to identify experts, how to elicit judgments and how to manage their interactions that have been shown to reduce social influence effects and increase collective accuracy and calibration of judgements in other settings. To what extent similar effects can be achieved in peer review is an empirical question that remains unaddressed. In our previous work, expert elicitations were generally constrained to a simple two-round process, but in its application to peer review, this may have to be extended to involve any number of rounds to allow additional consideration of authors’ responses upon resubmission. Moreover, there will be translational challenges in implementing structured expert elicitation protocols for the purposes of peer review, including the need to organise (at least for some manuscripts) synchronous discussion rounds between geographically (and time-zone) dispersed reviewers. Operationalising the above recommendations into everyday journal practices, across disciplines, will require some editorial bravery and careful experimentation.

## Data Availability

Not applicable.
